# Continuous Erector Spinae Plane Block for Pain Management in a Pediatric Kidney Transplant Recipient: A Case Report and Review of the Current Literature

**DOI:** 10.3390/jcm13041128

**Published:** 2024-02-17

**Authors:** Paolo Capuano, Gaetano Burgio, Serena Abbate, Giusy Ranucci, Kejd Bici, Davide Cintorino, Antonio Arcadipane, Gennaro Martucci

**Affiliations:** 1 Department of Anesthesia and Intensive Care, Istituto Mediterraneo per i Trapianti e Terapie ad alta Specializzazione (IRCCS-ISMETT), UPMCI (University of Pittsburgh Medical Center Italy), 90127 Palermo, Italy; pcapuano@ismett.edu (P.C.); gburgio@ismett.edu (G.B.); aarcadipane@ismett.edu (A.A.); 2 Pediatric Unit, Pediatric Center, Istituto Mediterraneo per i Trapianti e Terapie ad alta Specializzazione (IRCCS-ISMETT), UPMCI (University of Pittsburgh Medical Center Italy), 90127 Palermo, Italy; sabbate@ismett.edu (S.A.); granucci@ismett.edu (G.R.); 3 Surgical Unit, Pediatric Center, Istituto Mediterraneo per i Trapianti e Terapie ad alta Specializzazione (IRCCS-ISMETT), UPMCI (University of Pittsburgh Medical Center Italy), 90127 Palermo, Italy; kbici@ismett.edu (K.B.); dcintorino@ismett.edu (D.C.)

**Keywords:** fascial plane blocks, erector spinae plane block, kidney transplantation

## Abstract

Pain management in patients undergoing kidney transplantation requires careful consideration due to their altered physiology, and potential risks associated with certain analgesic options. In recent years, personalized and multimodal approaches have proven to be pivotal in perioperative pain management, as well as in children. Implementing regional analgesia methods offers a valuable solution in many pediatric surgical settings and the erector spinae plane block (ESPB) could represent a possible analgesic strategy in pediatric patients undergoing renal transplantation. Here, we report the case of a 13-year-old child who underwent living-donor kidney transplantation (LDKx) and received continuous erector spinae plane block (ESPB) for perioperative pain management. This multimodal approach with continuous ESPB resulted in optimal pain control without the need for opioids, allowing for early mobilization and for an optimal postoperative course.

## 1. Introduction

Kidney transplantation is the most effective treatment for children affected with end-stage renal disease [1]. When available, living-donor kidney transplantation (LDKx) carries several advantages, principally when performed as a pre-emptive strategy. These recipients often experience improved quality of life, better growth and development, and a reduced risk of complications associated with dialysis. Furthermore, having a kidney from a living donor tends to offer a longer lifespan for the transplanted organ due to less ischemic injury to the organ and lower rates of rejection [2].

Designated transplant centers for LDKx should construct multidisciplinary approaches to optimize outcomes and mitigate potential risks associated with the surgery and the hospital stay [3]. Pain is one of the main complications during the hospital stay for children because of its impact on general recovery and inflammation, and to the stress it causes for the family and caregivers. In cases of kidney disease, there are further challenges in pain management since impaired kidney function significantly limits the array of available analgesic options due to potential toxicity and altered drug metabolism related to alterations in drug distribution volume, modifications in protein binding, and delayed clearance [4]. In recent years, personalized and multimodal approaches have proven to be pivotal in perioperative pain management, as well as in children [5]. Implementing regional analgesia methods offers a valuable solution in many pediatric surgical settings; however, the use of a central regional anesthesia technique such as epidural blockade has been traditionally limited by concerns about bleeding—either primary or secondary to combining regional analgesia and anticoagulants after transplantation.

Today, a new generation of regional anesthesia techniques, called “fascial plane blocks”, are emerging as an effective alternative to conventional techniques such as paravertebral, epidural, or spinal blocks [6]. The primary target of fascial plane blocks is the deep fascia, a dense membrane of connective tissue that extends throughout the body. It surrounds and encases muscles, nerves, and other structures, including mechanoreceptors and nervous fibers [7].

These blocks, by avoiding direct injection into the nerve or toward the neural axis, can minimize the risk of serious complications such as neural injury and neuraxial hematoma and can potentially represent a valid alternative option in high-risk patients.

However, despite the use of fascial plane blocks becoming increasingly widespread even in the context of high-risk surgeries such as cardiothoracic surgery, there are still no specific guidelines on their use and management in patients at risk of bleeding [8].

Here, we report the case of a 13-year-old child who underwent LDKx and received continuous erector spinae plane block (ESPB) for perioperative pain management.

The parents of the child gave their consent for anonymous publication of the case report.

## 2. Case Presentation

A 13-year-old boy, with a weight of 38 kg and height of 152 cm, suffering from renal failure due to a dysplastic single kidney was referred to our institute for work up for a kidney transplant. The mother was identified as a possible compatible donor.

Blood creatinine was 6.8 mg/dL, BUN was 158 mg/dL, while daily urine output was still 2500 mL. Platelet count was 325 (✕10^3^/uL), and coagulation values were in the normal range.

In November 2023, the patient was scheduled for a pre-emptive kidney transplant from a living donor. General anesthesia was achieved according to standard practice (fentanyl 100 mcg, propofol 120 mg, cisatracurium 8 mg), including the monitoring of neuromuscular blockade and the depth of anesthesia with Bispectral Index (BIS), blood pressure monitoring via radial artery, and placement of a central venous catheter.

**Figure 1:**
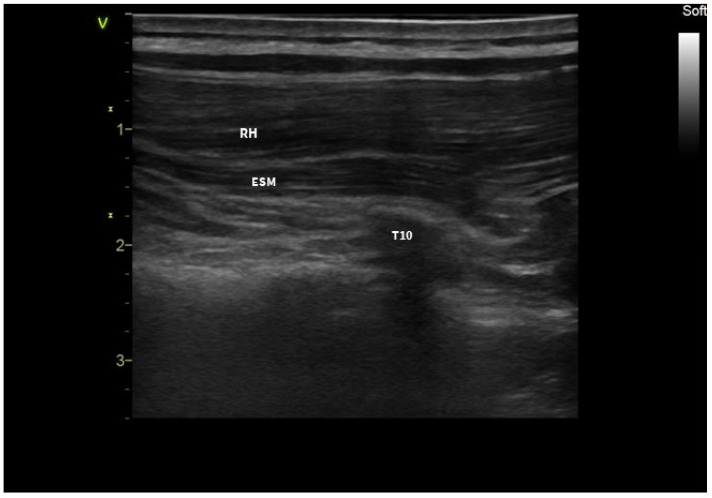
Sono-anatomy of the erector spinae plane block; RH: rhomboid muscle; ESM: erector spinae muscle; T10: transverse process.

After induction of anesthesia, the patient was placed in the left lateral position, and ultrasound-guided ESPB was performed for perioperative analgesia. A linear ultrasound transducer was placed on the parasagittal plane about 2 cm lateral to the T9-T10 transverse process and rhomboid major, and erector spinae muscles were identified superior to the hyperechoic transverse process (Figure 1).

**Figure 2:**
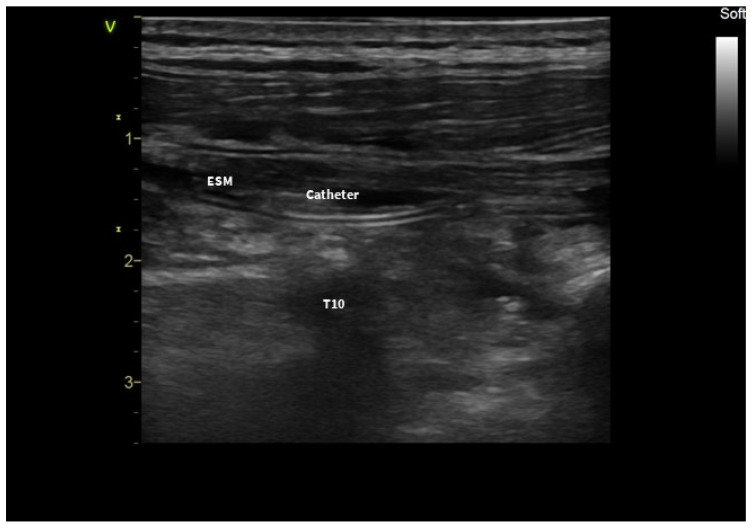
Ultrasound visualization of the ESPB catheter; ESM: erector spinae muscle; T10: transverse process.

Using an in-plane approach, an 18-gauge, 100 mm Contiplex Ultra 360^®^ needle (B.Braun, Melsungen, Germany) was inserted in the caudal–cephalad direction, with the tip of the T10 transverse process below the erector spinae muscle as the endpoint for the needle tip. After hydrodissection with 2 mL of normal saline, 20 mL of 0.375% ropivacaine was injected into the area. Then, a peripheral nerve catheter was inserted into the fascial plane, and placement was checked with ultrasound (Figure 2).

The operation lasted 3 h, and a hockey-stick incision into the right iliac fossa was performed for retroperitoneal access to the iliac vessels. Apart from the fentanyl administered at induction, no additional analgesics drugs were used. The patient was extubated in the operating room and then transferred to the intensive care unit for postoperative monitoring. He was moved to the surgery ward on postoperative day one.

Postoperative analgesia was achieved with paracetamol 500 mg every 6 h, together with ropivacaine 0.2% 20 mL every 8 h. With this multimodal approach, consistent good-quality analgesia (NRS < 3) was achieved, without side-effects such as constipation and postoperative nausea and vomiting. Postoperative physiotherapy was started immediately, and the patient was positioned out of bed on the second day and began walking early (Table 1).

In the first postoperative day, preventive anticoagulant treatment was initiated as per protocol (unfractionated heparin in continuous infusion in the range of 2–5 units/kg/h) until the fourth postoperative day, with no adverse effects observed.

Overall, the postoperative course was uneventful, complicated only by a urinary tract infection on day 8, promptly treated with piperacillin/tazobactam, and the patient was discharged on postoperative day 14.

## 3. Discussion

Pain management in patients with chronic kidney disease, particularly those who have undergone kidney transplantation, requires careful consideration due to their altered physiology, and potential risks associated with certain analgesic options. Drug metabolism and elimination can be significantly affected, leading to potential toxicity concerns with many pain medications.

Generally, morphine is still the most used drug for postoperative pain control, though its use, like other derivative opioids, is not without risk, because of the accumulation of toxic metabolites [9]. On the other hand, the use of other drugs such as gabapentinoids and NSAIDs, commonly administered in multimodal approaches, is also inadvisable in patients with chronic renal failure.

Consequently, the use of fascial plane blocks can be considered as a potential alternative in nephropathic patients, providing a valuable option for a multimodal approach to pain management.

The ESPB is a relatively new technique in the field of regional anesthesia and pain management. It was first described by Forero in 2016 and has gained attention in several surgical settings. In fact, the erector spinae is made up of three muscles (Spinalis, Longissimus, and Iliocostalis) that run from the sacrum to the skull, extending throughout the lumbar, thoracic, and cervical regions [10].

ESPB has gained attention and sparked debates over its mechanism of action since its first description. According to cadaveric studies and magnetic resonance imaging, it has been observed to provide analgesia for both somatic and visceral pain [11,12].

By injecting the local anesthetic into the interfascial plane between the erector spinae muscle and the transverse process, it can spread through channels in the intertransverse connective tissues. This spreading allows the local anesthetic to reach the ventral and dorsal rami of the thoracic spinal nerves, as well as the sympathetic ramus communicans at the intervertebral foramen level.

Furthermore, the involvement of lateral cutaneous branches of intercostal nerves is mentioned, suggesting that these nerve branches also contribute to the analgesic effect of the block [13].

ESPB has recently been described as a possible analgesic strategy in adult patients undergoing renal transplantation. When performed at the T9-10 level, ESPB provides analgesia without motor blocks in the abdominal–pelvic region: in the present case, a decreased sensation to pinprick and analgesia from T7 to T12 was recorded. In fact, ESPB provides both somatic and visceral analgesia by blocking both dorsal and ventral rami of the spinal nerves, and because of the transforaminal spread of local anesthetic into the paravertebral space and a variable amount of epidural spread [10].

In the 2019, Temirov et al. [14] first reported the successful use of a multimodal approach with a single ESPB shot in a 36-year-old man who underwent kidney transplantation. Continuous ESPB has also been described in the adult population for postoperative management in kidney transplantation. In a case series of 28 patients, Sharipova et al. [15] reported less pain and less opioid consumption, together with a lower incidence of nausea and vomiting, in 14 patients treated with continuous ESPB. 

Similar results were reported by Vishwanath et al. [16]: in their quality improvement project, they switched from epidural catheters to erector spinae plane catheters in managing postoperative pain in 13 kidney transplantations. They reported a better safety profile, minimal use of opioids, and lesser adverse effects.

Though the use of the ESPB in children has been described for various types of surgery [17,18,19,20], to the best of our knowledge, this is the first report describing the use of continuous ESPB for pediatric kidney transplantation.

The importance of minimizing opioid use during the perioperative period for kidney transplant recipients has recently been addressed in the literature. As known, Enhanced Recovery After Surgery (ERAS) protocols focus on multimodal analgesia strategies aimed at reducing opioid consumption in various surgical settings, including kidney transplantation [21]. Therefore, adopting alternative or complementary analgesic strategies becomes crucial in mitigating opioid-related risks, and improving postoperative outcomes.

In the present case, a multimodal approach with continuous ESPB resulted in optimal pain control without the need for opioids, thus allowing for early mobilization. By eliminating the need for opioids, we observed a rapid recovery of intestinal function, without side-effects such as constipation and vomiting. Though desirable in all patients, a rapid and regular course is particularly desirable in pediatric cases, in which it is necessary to try to eliminate as much trauma as possible. Moreover, ESPB demonstrated a good safety profile, despite the need to initiate anticoagulant therapy with heparin, and no complications were observed. This is consonant with what is reported in the literature regarding the efficacy and the safety of fascial plane blocks in patients at high risk of bleeding in the cardiothoracic setting [8,22]. Specifically, Toscano et al. investigated the safety of fascial plane blocks, specifically continuous ESPB and SAPB in patients receiving anticoagulation and coagulopathy. They analyzed 70 patients undergoing minimally invasive mitral valve surgery through a right mini-thoracotomy. These patients received either continuous ESPB or SAPB for perioperative pain control. No adverse outcomes attributable to SAPB or ESPB in terms of vascular puncture, active bleeding, or hematoma formation were reported. 

In fact, one of the advantages often highlighted with the Erector Spinae Plane Block is its anatomical location, which is deep in the erector spinae muscle plane and superficial to the transverse processes. This positioning is thought to contribute to a reduction in certain risks when compared to other regional anesthesia techniques [22].

Due to the distance of the ESP from major vessels and the spinal cord (medulla), there is a decreased risk of complications such as hypotension and hematoma when compared to techniques like Thoracic Epidural Analgesia (TEA) and Paravertebral Block (PVB) [8]. 

Furthermore, thanks to the interforaminal spread of the injectate in the ESPB, the risk of pneumothorax is reduced compared with PVB, where the needle is advanced closer to the pleura [22].

These anatomical considerations are also in line with a recent review that analyzed the safety and risk profiles of thoracic PVB and ESPB in patients receiving anticoagulant or antiplatelet therapy for cardiothoracic surgery or thoracic procedures [23]. The authors analyzed 15 articles and evidenced a low risk of bleeding associated with PVB and minimal or absent risk for ESPB, suggesting their favorable safety profiles for patients receiving anticoagulant or antiplatelet therapy, particularly in the context of cardiothoracic surgery or thoracic procedures.

Similar considerations were made by a panel of Canadian experts on regional anesthesia: they reviewed the evidence and classified the risk of bleeding complications following regional nerve blocks [24]. The ESPB was considered low-risk.

In the present case, the ESPB catheter was removed without stopping UFH infusion and no complications were observed. 

There are currently no specific guidelines available on the management of the ESPB catheter during anticoagulation therapy. However, Adhikary et al. [25] reported the use of continuous ESPB for pain management in five patients undergoing left thoracotomy for left ventricular assist device placement. Despite the need for prolonged postoperative heparinization, they reported no complications in the management of catheters.

Similarly, in the study by Toscano et al. [8], regional catheters were removed at 48 hours irrespective of the international normalized ratio (INR) value and no complications were reported.

Finally, in the context of a multimodal approach in renal surgery, a possible alternative is represented by the quadratus lumborum block (QLB), which targets the somatic and visceral fibers on the anterolateral abdominal wall, achieving sensory block [26].

Onay et al. recently compared QLB and ESPB in terms of their effects on postoperative pain in open nephrectomy: they found that both approaches achieve similar results for at-rest and at-movement pain scores and opioid consumption during the postoperative period [27].

In our opinion, QLB is a valid option for pain management in kidney surgery but has some limitations in the field of renal transplantation. First, the QLB is considered a deeper block at high risk of bleeding, with a needle trajectory into a noncompressible space. Consequently, the risk of bleeding and complications is similar to that of the lumbar plexus block. Moreover, the postoperative catheter placement is probably more comfortable with the ESPB, with less impediment regarding early mobilization.

## 4. Conclusions

Continuous ESPB appears to be a valid and safe option in the multimodal analgesia of patients undergoing renal transplantation, even in the pediatric population, allowing good analgesia with a sparing of opioids. Future randomized trials will be needed to confirm our preliminary report.

## Figures and Tables

**Table 1:** Ropivacaine administration, lab values, and progress indicators in the postoperative period.

	Operative Day	POD 1	POD 2	POD 3	POD 4
	**Catheter placement**				
**Ropivacaine dose**	0.375% 20 mL pre-operative	0.2% 20 mL × 3	0.2% 20 mL × 3	0.2% 20 mL × 3	Catheter removed
**NRS at rest**		0	0	0	0
**NRS at movement**		3	2	0	0
**Gas canalization**		x	x	x	x
**Stool canalization**			x	x	x
**Creatinine mg/dL**	6.8	2.52	1.2	1.21	0.69
**Platelet (×10^3^/uL)**	325	271	285	299	301
**INR**	1.03	1.01	1	0.98	0.95
**Diet**		Sips of water	Soft diet	Regular diet	Regular diet
**Movement**		In the bed	Out of bed	Ambulating	Regular activity

## Data Availability

Data are contained within the article.
